# The emerging role for metabolism in fueling neutrophilic inflammation

**DOI:** 10.1111/imr.13157

**Published:** 2022-11-03

**Authors:** Tyler Morrison, Emily R. Watts, Pranvera Sadiku, Sarah R. Walmsley

**Affiliations:** ^1^ University of Edinburgh Centre for Inflammation Research, Queen's Medical Research Institute, University of Edinburgh Edinburgh UK

**Keywords:** immunometabolism, inflammation, neutrophil, trained immunity

## Abstract

Neutrophils are a critical element of host defense and are rapidly recruited to inflammatory sites. Such sites are frequently limited in oxygen and/or nutrient availability, presenting a metabolic challenge for infiltrating cells. Long believed to be uniquely dependent on glycolysis, it is now clear that neutrophils possess far greater metabolic plasticity than previously thought, with the capacity to generate energy stores and utilize extracellular proteins to fuel central carbon metabolism and biosynthetic activity. Out‐with cellular energetics, metabolic programs have also been implicated in the production of neutrophils and their progenitors in the bone marrow compartment, activation of neutrophil antimicrobial responses, inflammatory and cell survival signaling cascades, and training of the innate immune response. Thus, understanding the mechanisms by which metabolic processes sustain changes in neutrophil effector functions and how these are subverted in disease states provides exciting new avenues for the treatment of dysfunctional neutrophilic inflammation which are lacking in clinical practice to date.

## 1 INTRODUCTION

Neutrophils respond rapidly to infection and injury, providing a critical first line of host defense. To do so, they employ a range of specialized functions which include the ability to phagocytose pathogens, release cytokines and cytotoxic mediators, and regulate other elements of the immune response. They are short‐lived cells, undergoing constitutive apoptosis and, as such, are in a state of high turnover. Each day large numbers are released into the blood where they circulate before being recruited to tissues in response to infection or injury signals. Here, they sense tissue injury and pathogens to mount a proportionate response. These sites may be metabolically challenging and neutrophils are adapted to sense and adapt their metabolic program, enabling them to continue to function in these hostile environments.

More broadly, the last decade has seen an explosion in our understanding of the role for metabolism in regulating immune cell function in both health and disease. Far beyond the simple provision of energy, cellular metabolites enable post‐translational modifications, epigenetic regulation of transcription and can themselves act as signaling molecules. However, given their short lifespan, technical challenges, and historic misconception of their simplicity, the role of metabolism in defining neutrophil function is only recently gaining interest. This review will outline the major metabolic pathways employed by neutrophils, summarize the role of the mitochondria in neutrophil function, and explore how altered neutrophil metabolism contributes to disease.

## 2 PECULIARITIES OF NEUTROPHIL METABOLISM

**FIGURE 1:**
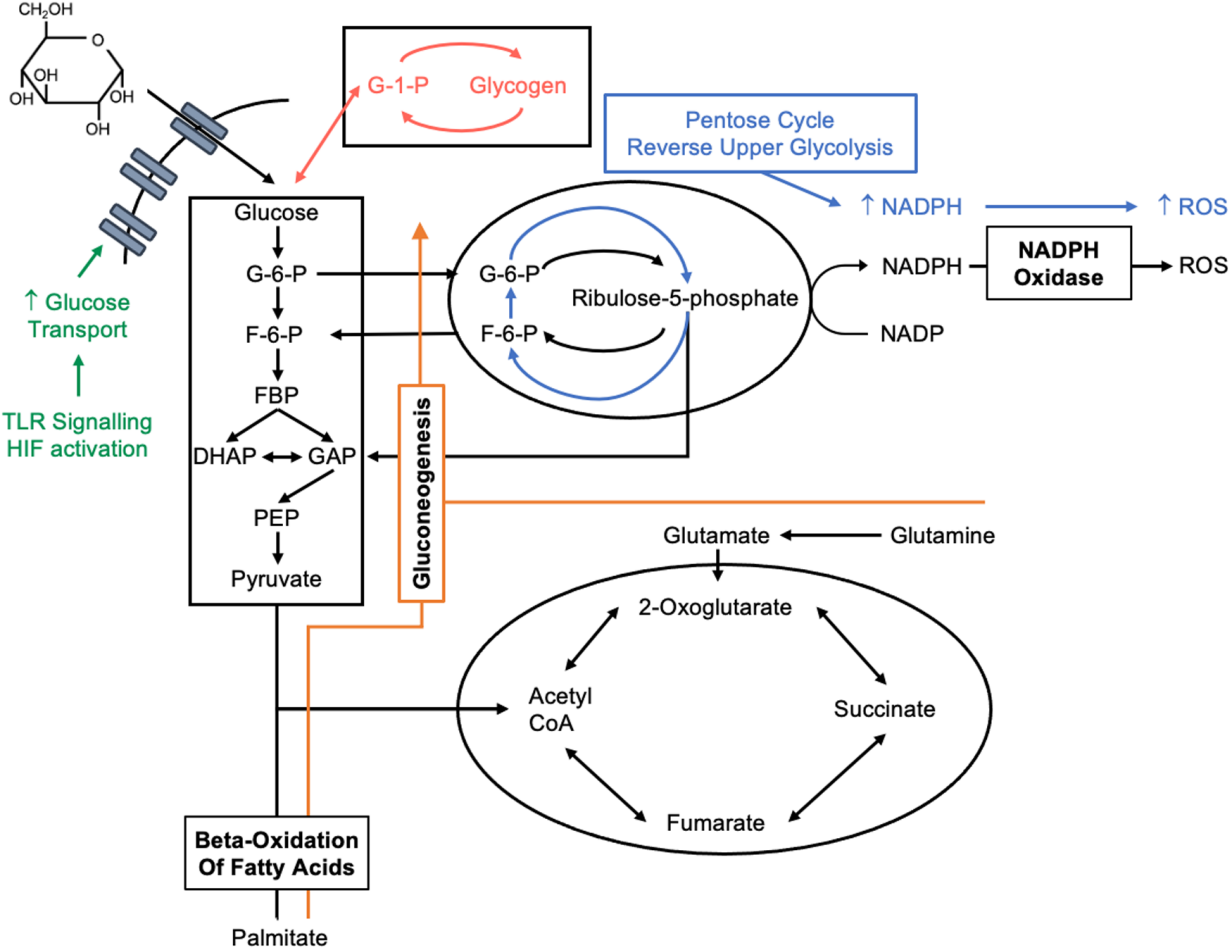
Neutrophils are predominantly glycolytic but can alter their metabolic program in response to environmental signals, energetic demands, and nutrient availability. Glucose transport is upregulated in response to TLR activation and HIF signaling (depicted in green). Glutamine and fatty acids can both be utilized to replenish central carbon metabolism (depicted in orange). Glycogen can be both synthesized and broken down as required to provide energy storage when glucose is abundant and substrate when glucose is limited (depicted in red). Following activation with multiple different stimuli, neutrophils reconfigure metabolism to maximize ROS production via a move to a pentose cycle, supported by reverse upper glycolysis (Depicted in blue). G‐1‐P, glucose‐1‐phosphate; G‐6‐P, glucose‐6‐phosphate; F‐6‐P, fructose‐6‐phosphate; FBP, fructose‐1,6‐bisphosphate; DHAP, dihydroxyacetone phosphate; GAP, glyceraldehyde 3‐phosphate, PEP, phosphoenolpyruvate

Mammalian cells derive energy from 3 key sources: carbohydrate, protein, and fats (Figure 1). The breakdown of these substrates ultimately leads to the generation of adenosine triphosphate (ATP), the cellular energy currency. Protein and fats enter the mitochondrial tricarboxylic acid cycle (TCA) and electron transport chain during catabolism in a process that is oxygen dependent. The breakdown of the key carbohydrate glucose also requires mitochondrial oxygenation for complete efficiency. However, in contrast to other substrates, ATP can nevertheless be generated by glycolysis in oxygen deplete environments. Oxygen exists in arterial blood at 10‐12 kPa, reducing to 4‐5 and 2 kPa in venous blood and skin, respectively, although the precise availability is tissue‐dependent. During inflammation, factors such as edema, microthrombi, and reactive oxygen species contribute to the development of localized hypoxia and nutrient insufficiency. In addition, recruitment of metabolically active cells increases oxygen consumption with oxygen availability at inflammatory sites estimated to be <1 kPa.^1^ Neutrophils, therefore, not only contribute to this hostile environment but must be sufficiently resilient to function within it.^2^ Consequently, neutrophils possess the metabolic adaptations to utilize glycolysis to supply over 90% of their ATP, irrespective of oxygen availability, a response augmented by activation of the transcription factor hypoxia‐inducible factor (HIF). Although less efficient that oxidative metabolism (in terms of absolute amount of ATP produced per molecule of glucose), this glycolytic phenotype ensures ongoing access to ATP, independent of oxygen availability. It also allows rapid access to ATP, enabling neutrophils to meet the sudden increase in energy demands required for effective antimicrobial defense including chemotaxis, degranulation, phagocytosis, and respiratory burst activity.^3^, ^4^

This reliance on glycolysis generates a further challenge for these resourceful cells. Sites of inflammation are frequently characterized by limited nutrient availability, including low glucose levels.^5^ Neutrophils have a number of strategies to overcome this challenge. Firstly, circulating human neutrophils have evolved the ability to rapidly enhance glucose uptake from the extracellular environment following activation of toll‐like receptors (TLRs) through enhanced translocation of glucose transporters to the cell surface.^6^ Neutrophils also have the capacity to store glucose in the form of glycogen, which they can utilize to fuel inflammatory responses when access to extracellular glucose is limited.^7^, ^8^ Of interest is the recent finding that both human and murine neutrophils possess an active gluconeogenic pathway, a process originally considered to be confined to hepatocytes.^8^ This provides the neutrophil with the ability to use non‐glucose substrates to form both glucose and glycogen and is now known to play an important part in fueling effective neutrophil responses in the tissues,^5^, ^8^ opening new avenues for exploration of neutrophil biology. In dendritic cells, glucose and glycogen‐derived carbons have been shown to contribute to distinct metabolic pathways with consequences for cellular functions.^9^ Whether neutrophils exhibit similar functional segregation of carbons, depending on their metabolite source, remains unknown. There is early evidence that glucose compartmentalization can regulate neutrophil function: Patients with glycogen storage disease type Ib lack the glucose‐6‐phosphate transporter, and thus, endoplasmic reticulum (ER) cycling of glucose fails to take place. As a result, glucose uptake is diminished, which simultaneously impairs glucose utilization and promotes both ER and mitochondrial oxidative stress, ultimately regulating neutrophil survival.^10^ For inflammatory neutrophils, the capacity to switch between these metabolic programs provides these cells with an armory of metabolic responses which they can engage to sustain biosynthetic activity when oxygen and nutrients are limited.

Glucose not only contributes to neutrophil energy homeostasis but plays an essential role in antimicrobial responses against certain pathogens.^11^ Diversion of glucose‐6‐phosphate through the oxidative pentose phosphate pathway (PPP) is essential for NADPH generation which reduces oxygen to form reactive oxygen species (ROS) through the NADPH oxidase (NOX) system. Interesting recent work has demonstrated that human and murine neutrophils rapidly enhance NADPH production when demand is present through generation of a pentose cycle and reverse upper glycolysis.^12^ One glucose molecule therefore contributes up to six NADPH, as opposed to two through the canonical PPP, permitting rapid activation of the cell's effector functions. ROS generation through NOX is a cornerstone of an effective neutrophil response, as highlighted by the infectious disease burden in patients with chronic granulomatous disease who lack NOX activity.^13^ ROS ultimately leads to hypochlorous acid generation which is one element of the antimicrobial arsenal.^14^ However, ROS and therefore NOX and NADPH also play diverse roles in the regulation of neutrophil activity through intracellular signaling. This can occur through modification of the redox state of target proteins or lipids^15^ and can regulate key processes such as chemotaxis through modulation of phosphatase activity in studies of murine bone marrow neutrophils.^16^ Finally, while increasing complexity has been appreciated in the formation of NETs to a diverse range of stimuli, glucose utilization is central to phorbol 12‐Myristate 13‐Acetate (PMA)‐induced NET formation: While glycolysis is necessary for fueling PMA‐induced NET formation,^17^ the PPP appears to be essential for the ROS which ultimately determines the decision to form NETs in human peripheral blood neutrophils.^18^ The PPP may then not only fuel oxidative antimicrobial responses but play an important role in intracellular ROS signaling and instruction of neutrophil fate.

## 3 NEUTROPHIL MITOCHONDRIAL METABOLISM

**FIGURE 2:**
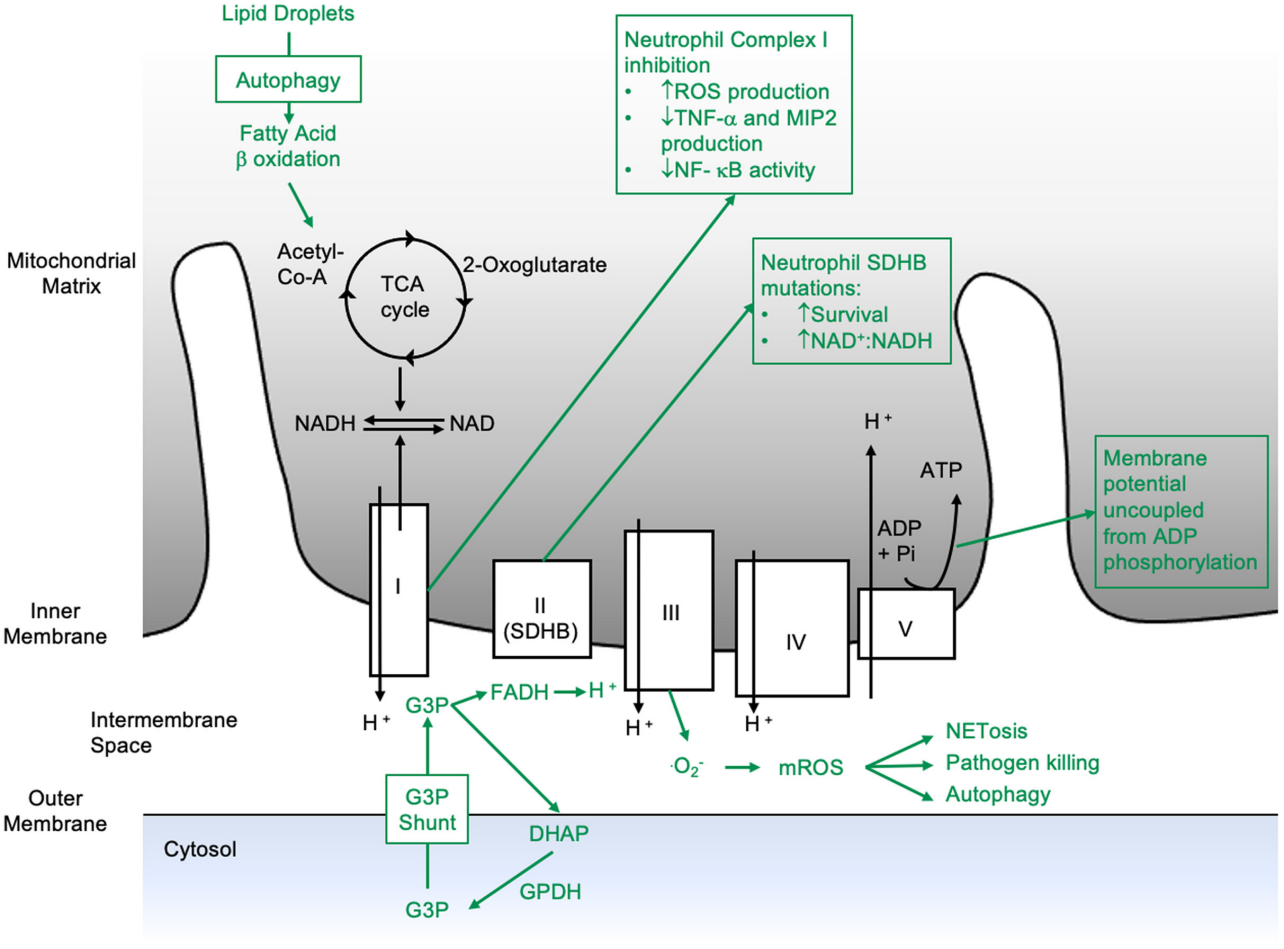
Although the electron transport chain is dispensable for ATP synthesis in neutrophils, mitochondria remain essential for a wide range of functions. Those which have been shown to be of relevance in neutrophils are highlighted in green. SDHB, succinate dehydrogenase B; G3P, glycerol‐3‐phosphate; DHAP, dihydroxyacetone phosphate; GPDH, glycerol‐3‐phosphate dehydrogenase

Despite their reliance on glycolysis over oxidative respiration, neutrophils do contain mitochondria. The role of the neutrophil mitochondria is somewhat controversial and although there is a long‐held view that their key role is in the regulation of apoptosis,^19^ increasing evidence has demonstrated an important role in coordinating neutrophil metabolic responses (Figure 2). Indeed, although neutrophil mitochondria are morphologically indistinct in comparison with other cells they do, nevertheless, possess a membrane potential.^19^ Interestingly, this membrane potential appears to be uncoupled from ADP phosphorylation, at least in quiescent human neutrophils.^20^

To what extent, and for what purpose, neutrophils utilize mitochondrial metabolism therefore remains an open question. In this regard, tissue nutrient availability is an important consideration. The enzymes of the TCA cycle and oxidative phosphorylation are important regulators of terminally differentiated neutrophil function and survival. Succinate dehydrogenase B (SDH) oxidizes succinate to fumarate in the TCA cycle while also functioning as complex II in the electron transport chain. In neutrophils from patients with reduced SDH activity, SDH activity was shown to play an important role in neutrophil survival, a result replicated with chemical inhibition.^21^ Similarly, through interrogation of mitochondrial complex I by rotenone, a role for electron transport chain activity has been identified in LPS‐mediated neutrophil activation and tissue injury in murine models, although this effect was independent of ATP generation.^22^

The role for specific substrates in sustaining neutrophil TCA, electron transport chain activity and the instruction of neutrophil function remains relatively scantly investigated. Neutrophils have been demonstrated to utilize the mitochondrial oxidation of glycerol‐3‐phosphate (G3P) in a so‐called G3P shuttle to maintain mitochondrial membrane potential. This G3P shuttle provides electrons to complex III and appears to play an important role in regenerating NAD+ for continuing glycolysis,^20^ and therefore, even for glycolytic metabolism, the neutrophil mitochondrion appears to play an important role. Moreover, in human blood neutrophils, this shuttle was shown to promote the generation of mitochondrial ROS, which regulated HIF1‐α stability and therefore neutrophil survival, phagocytosis, and protease release.^23^

In other cells, the complete oxidation of glucose through the TCA cycle is an important source of ATP. However, little evidence exists to suggest that glucose‐derived acetyl CoA is an important mitochondrial substrate in neutrophils. Fatty acid‐derived acetyl CoA, which enters the TCA cycle to fuel electron transport chain activity, represents an alternative mitochondrial substrate. In mature neutrophils, a small role for fatty acid oxidation in ATP production in human peripheral blood neutrophils has been demonstrated through inhibition by etomoxir.^8^ However, the importance of this in terminally differentiated neutrophils remains to be fully explored. Fatty acid oxidation likely plays a much greater role in immature neutrophils and in specific neutrophil subsets and is discussed below along with other facets of neutrophil lipid metabolism.

The amino acid glutamine is highly abundant and may contribute both to biosynthetic pathways such as protein synthesis and to catabolic pathways. The breakdown of glutamine through glutaminolysis leads to the formation of alpha‐ketoglutarate which feeds into the TCA cycle. Through heavy carbon labeled experiments, human neutrophils have been shown to metabolize glutamine,^8^, ^24^ with the downstream metabolic intermediaries contributing to multiple important pathways and functions: Glutaminolysis leads to NADPH production,^25^ generating substrate for neutrophil NOX and therefore oxidative burst. Furthermore, glutamine supports neutrophil NET formation in response to PMA which is known to be dependent on ROS.^17^, ^26^ Interestingly, <1% of glutamine used by inflammatory peritoneal rat neutrophils becomes fully oxidized,^27^ suggesting additional roles for glutamine beyond ROS production. In this regard, glutamine is an important substrate for biosynthetic reactions including that of glutathione and nucleotide synthesis. However, an in‐depth interrogation of these pathways has yet to take place in neutrophils. Glutamine has been shown to support neutrophil central carbon metabolism^5^, ^8^ particularly in conditions of nutritional stress. Moreover, this supports a hyperinflammatory phenotype in murine lung neutrophils. Of considerable interest is the observation that glutamine also supports neutrophil gluconeogenesis, which ultimately leads to the de novo formation of glycogen.^8^ Glycogenolysis of newly formed glycogen via a glycogen shunt has been shown to have specific roles in early metabolic reprogramming during dendritic cell activation. Human blood neutrophils have been shown to utilize different sources of glucose for different functions, with chemotaxis preferentially utilizing glucose taken up from the extracellular environment while phagocytosis utilizes glycogenolysis.^28^

As our understanding of the complexity and heterogeneity of the neutrophil population expands, it has become clear that metabolic programs also vary with the stage of neutrophil differentiation. Specifically, while glycolysis is the principal energy source in mature neutrophils, oxidative metabolism appears to play a greater role in immature neutrophils. One source of substrate for oxidative metabolism is via autophagy, where cytosolic components become enclosed in double‐membraned vesicles termed autophagosomes. Through recruitment of lysosomes, cellular elements are recycled, clearing damaged components and providing an energetic source. During differentiation, a progressive shift from oxidative phosphorylation to glycolysis occurs, with generation of fatty acids by autophagy of lipid droplets critical to immature neutrophil energy status and progression of differentiation in the mouse bone marrow compartment.^29^ In the absence of lipolysis, provision of pyruvate or free fatty acids restored energy reserves and differentiation, demonstrating the importance of mitochondrial ATP provision from fatty acid oxidation to immature neutrophil development.

Beyond cellular energetics, ATP via purinergic signaling has emerged as an important regulator of neutrophil function. Although in the mature neutrophil, mitochondrial ATP provision is low, it has an interesting role in the regulation of chemotaxis in human and murine neutrophils. During chemotaxis, ATP is released at the leading edge and signals via purinergic receptors in an autocrine manner.^30^ Studies of human neutrophils have demonstrated that collapse of neutrophil mitochondrial membrane potential by FCCP inhibited chemotaxis (although did not alter respiratory burst or phagocytosis).^31^ Furthermore, it was demonstrated that mitochondria were responsible for the provision of the ATP, via mTOR signaling, in response to the chemoattractant fMLP in human neutrophils. Finally, defective chemotaxis has been observed in inflammatory neutrophils of mice lacking purinergic receptors.^32^ The cellular source of ATP is therefore important in regulating cellular activity and even a small ATP contribution may have a significant impact on inflammatory outcomes.

Electron transport chain activity in the mitochondrion, particularly complex III, also leads to the generation of mitochondrial ROS (mROS).^33^ mROS plays several important roles in the cell. For example, in macrophages, a loss of uncoupling protein 2 increased mROS production and led to more efficient elimination of the protozoa *Toxoplasma gondii* through MAPK activation.^34^, ^35^ Additionally, accumulation of mROS was important for the initiation of autophagy during starvation in cell lines,^36^ which may be especially pertinent to the neutrophil's biosynthetic response to nutrient‐deprived sites.^5^ The role of ROS in NET formation has also gathered considerable interest with several pathways now described from diverse stimuli. In NADPH‐independent NETosis, of human blood neutrophils, mROS was necessary.^37^, ^38^ The substrates and metabolic switches that ultimately guide mROS formation during neutrophil responses require further exploration.

Finally, not only do mitochondria play a role in regulating chromosomal DNA expulsion during NET formation, they can also form NETs themselves through the release of mitochondrial DNA (mtDNA) from live human blood neutrophils.^39^ NETs composed of mtDNA were detected in patients following trauma,^40^ although their role remains unclear as they lack histones, considered to be key in the antimicrobial activity of NETs. mtDNA in this context may instead play a role in precipitating the inflammatory response through activation of inflammatory pathways.^41^ Given their relatively short lifespan and low reliance on mitochondrial ATP, the neutrophil may be uniquely placed to expulse mitochondria for such purposes.

## 4 LIPID METABOLISM: CELLULAR ENERGETICS TO SIGNALING MOLECULES

Beyond their role in cellular energetics, lipids are also important for multiple cellular functions. Lipids constitute a large and diverse class of biomolecules, reflected in the multitude of roles they play in the cell^42^ (Figure 2).{Fahy, 2011 #1} They can be classed into five major subcategories: free fatty acyls (eg palmitic acid and oleic acid), glycerolipids (eg triacylglycerols), glycerophospholipids (eg phosphatidylcholine, phosphatidylserine), sterol lipids (eg cholesterol), and sphingolipids (eg sphingomyelin). Fatty acids can be stored in the form of triacylglycerols in lipid droplets. Triacylglycerols (TG) can then be broken down by lipolysis and the resulting fatty acids can enter beta‐oxidation to contribute to both ATP generation and reducing power, in the form of NADPH. Glycerophospholipids, sphingolipids, and sterols primarily serve as building blocks for cellular and organelle membranes.^43^ In addition, precursors of bioactive lipid mediators known as polyunsaturated fatty acids (PUFA) are released from membrane phospholipids in response to cytokines during early phases of inflammation and infection. Arachidonic acid (AA) is a common endogenous precursor released from membrane phospholipids by cytosolic phospholipase A2 and transformed into numerous potent bioactive eicosanoids: prostaglandins, leukotrienes (LT), and lipoxins.^44^

It is widely appreciated that such lipid mediators play a critical role in initiating leukocyte migration required for host defense.^45^ Leukotrienes and prostaglandins are the main eicosanoids produced following neutrophil activation and result in the induction of the acute inflammatory response. AA is converted to prostaglandin H_2_ (PGH_2_) by cyclo‐oxygenase 2 and is further metabolized into active prostaglandin E_2_ (PGE_2_).^46^ Human neutrophils express high levels of 5‐lipoxygenase, the enzyme catalyzing the conversion of arachidonic acid to 5‐hydroperoxyeicosatetraenoic acid (5‐HPETE) and leukotriene A_4_ (LTA_4_).^47^ On stimulation, 5‐lipoxygenase translocates to the nuclear membrane where it co‐localizes with 5‐lipoxygenase‐activating protein and cytosolic phospholipase A2, allowing efficient leukotriene production. LTA_4_ is converted to leukotriene B_4_ (LTB_4_) by LTA_4_ hydrolase.^48^ LTB_4_ is a potent chemoattractant of neutrophils, as well as inducing adhesion to endothelial cells, degranulation, and superoxide production.^49^, ^50^, ^51^ The formation of leukotrienes is dependent on the endogenous and exogenous arachidonic acid pools. Endogenous AA is preferentially converted into LTB_4_ whereas exogenous AA contributes to LTA_4_ for export.^52^ Importantly, the proinflammatory lipid mediators PGE_2_ and PGI_2_ contribute to progression from acute to chronic inflammation in a murine model of collagen‐induced arthritis.^53^ Furthermore, redundancy is noted within this system between the multiple prostaglandin receptor types and subtypes. Lipid droplets serve as major organelles for the generation of AA‐derived eicosanoids.^54^ Triacylglycerols constitute the major lipid class of the neutrophil lipid droplet core,^55^ and resting human neutrophils have been shown to contain a high proportion of triacylglycerols in comparison with CD14^+^ monocytes and CD4^+^ T cells.^56^ Adipose triglyceride lipase (ATGL), which degrades triacylglycerols, directly regulates substrate availability for proinflammatory lipid mediator synthesis. In humans, mutations in ATGL lead to Jordan's anomaly, characterized by TG accumulation in many cells and tissues, including in circulating leukocytes. Studies in mice lacking ATGL in the myeloid compartment demonstrated that loss of ATGL in neutrophils results in reduced release of lipid mediators during an inflammatory response but also enhanced chemotaxis and some evidence of increased activation, highlighting the pleiotropic effects of lipid mediators.^57^ Interestingly, human neutrophils have been shown to take up high levels of fatty acids which, rather than undergoing oxidation (as was observed in lymphocytes), are incorporated into newly synthesized TG.^58^ These triglycerides are thought to act as a source of not just lipid mediators, but also fatty acid for phosphatidylcholine synthesis during phagocytosis.^59^ In keeping with this, LPS‐stimulated neutrophils have elevated levels of TG with increased amounts of palmitic acid.^60^ A recent study by our group supported these findings of increased fatty acid transport into neutrophils and high levels of palmitic acid entering into beta‐oxidation.^8^

Alterations in lipid content have been linked to the regulation of human neutrophil function and survival. Both cholesterol depletion and sphingomyelin degradation result in increased NET formation.^61^ The membranes of LPS‐stimulated neutrophils have been shown to display increased fluidity measured by the cholesterol: phospholipid ratio.^60^ Lipid rafts have important biological functions related to membrane signaling and protein trafficking. Both cholesterol and sphingomyelin are involved in the formation of these highly ordered and tightly packed membrane microdomains^62^ which have been reported to play a role in neutrophil migration.^63^

Neutrophil apoptosis is preceded by the de novo synthesis of the bioactive sphingolipid ceramide. The accumulation of endogenous 16‐ and 24‐carbon atom ceramides which are not derived from sphingomyelin partially contributes to spontaneous human neutrophil apoptosis through the activation of caspases.^64^ These studies demonstrate a correlation between lipid abundance and neutrophil survival and function. However, further work is required to delineate the exact metabolic processes involved in the generation of these lipids, and the mechanisms by which alterations in their abundance might impact upon cell membrane fluidity and lipid mediator availability.

A link between phospholipid metabolism and neutrophil inner mitochondrial membrane integrity is well established. The phospholipid cardiolipin is almost exclusively found in the inner mitochondrial membrane. Tafazzin (TAZ) is an enzyme which processes the final step of cardiolipin maturation, replacing saturated with unsaturated acyl chains. Inherited mutations in the TAZ gene result in defects in cardiolipin synthesis and contribute to a disorganized inner mitochondrial membrane resulting in Barth syndrome.^65^ Barth syndrome is characterized by cardiomyopathy, skeletal muscle myopathy, and neutropenia. Although data suggest that the neutropenia is caused by an increased propensity for apoptosis as a result of ER stress,^66^ how inherited mutations in TAZ increase sensitivity to ER stress remains unknown. Cardiolipin is known to be critical in the stabilization of protein complexes such as the electron transfer chain I, III, and IV, as well as ATP synthase which ensures electron transfer chain efficiency.^67^ Changes in neutrophil membrane bilayer organization may result in destabilization of enzymes or enzyme complexes involved in energy production and may, in turn, result in generation of mROS but this has not yet been shown definitively.

**FIGURE 3:**
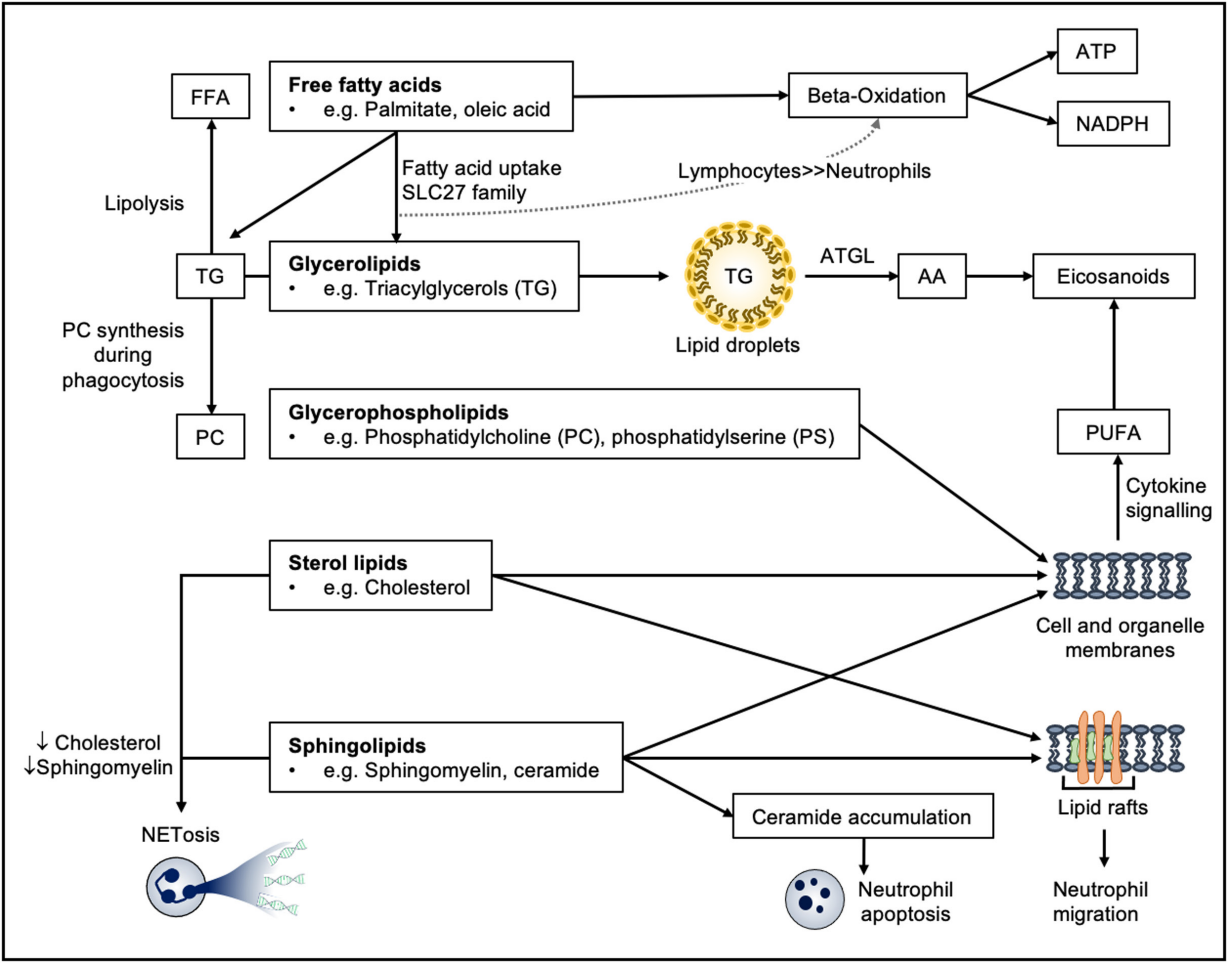
Lipids and their metabolites constitute a diverse group of mediators with equally diverse effects on neutrophil phenotype and function. Through both catabolic and anabolic programs, neutrophils can drive ATP synthesis, generate reducing power, synthesize downstream mediators such as eicosanoids, build cellular membrane components, cause NETosis and apoptosis and facilitate neutrophil migration. FFA, free fatty acids; AA, arachidonic acid; PUFA, polyunsaturated fatty acids

These studies demonstrate that lipid mediators act as key neutrophil signaling molecules, required for effective migration, recruitment, and phagocytosis (Figure 3), although often the specific molecular mechanisms involved remain poorly defined. The functions of membrane lipids which are not related to biologically active eicosanoids remain unknown. Areas for future research include investigation of how the cellular lipidome is regulated to sustain neutrophil function and survival, as well as how the organization and compartmentalization of lipids in the phospholipid bilayer relates to key neutrophil effector functions.

## 5 ONE CARBON METABOLISM, BIOSYNTHESIS, AND ANTIOXIDANT DEFENSE

Cell survival requires the flexibility to synthesize a multitude of molecules to meet demand as and when required. A key biosynthetic pathway is the one‐carbon (1C) metabolism pathway, centered around the metabolism of folate. Specifically, multiple sources of carbon including serine, glycine, and histidine feed into the interlinked folate and methionine cycles which transfers one carbon units to new molecules and ultimately leads to the formation of purines, pyrimidines, amino acids, and phospholipids. Additionally, the methylation or transfer of 1C units to DNA leads to modification of gene expression and therefore regulation of cellular function. Unsurprisingly, the focus for 1C metabolism has been in proliferating tissues with particular interest in development, hematopoiesis and in cancer cells. Indeed, inhibition of folate metabolism by methotrexate is an established therapeutic option in both cancer and immune‐mediated disease. More diverse roles for the regulation of T‐cell responses have been identified, where 1C metabolism regulated the formation of purines and thymidine, permitting proliferation and naïve T‐cell function. This response was mitochondria dependent and appeared to protect cells from DNA damage.^68^ Given the intrinsic link between 1C metabolism and proliferation, comparatively little attention has been directed toward the cycle's role in neutrophils.

However, this pathway has diverse roles in the cell which merit further consideration. Neutrophils are more biosynthetically active than was previously considered, and modify their metabolism based on their environment and needs to support this.^5^ While neutrophils have an active oxidative PPP to generate NADPH, they are terminally differentiated cells and therefore little consideration has been given to their need for ongoing nucleotide synthesis and the role non‐oxidative PPP in this. With evidence that redox balance regulates serine biosynthesis and the 1C pathway in cancer, much remains to be explored in the context of neutrophil anabolic reactions.^69^ It is interesting to consider to what extent a neutrophil requires new nucleotides and one could speculate that they may have a role in nucleotide salvage or mitochondrial biogenesis. Additionally, NET formation employs active DNA repair mechanisms^70^ which utilize the pentose phosphate pathway in other cell types.^71^ It will be interesting to examine the role that these previously overlooked biosynthetic pathways have in neutrophil homeostasis and in neutrophil‐mediated host defense and illness.

While the oxPPP is considered the dominant source of NADPH, an almost comparable role for folate metabolism was identified in a cell line model.^72^ Given the importance of NADPH to neutrophil ROS formation and pathogen killing, it is intriguing to consider to what extent 1C metabolism contributes to neutrophil antimicrobial responses. Moreover, in nutrient deplete conditions, the mitochondrial 1C pathway has been shown to be necessary for the de novo synthesis of glutathione and therefore essential in maintaining cellular redox balance.^73^ In murine neutrophils, the ability to form reduced glutathione was necessary to sustain the oxidative burst and antimicrobial killing.^74^ Moreover, depletion of glutathione has been suggested to be an important step in mouse bone marrow neutrophil apoptosis.^75^

The nuclear factor erythroid 2‐related factor 2 (Nrf2) is another important defense against redox stress. NRF2 is constitutively expressed transcription factor but targeted for proteasomal degradation by Kelch‐Like ECH‐associated protein 1 (KEAP1) under homeostatic conditions. During oxidative stress, KEAP1 is inactivated leading to NRF2 accumulation and its translocation to the nucleus where it regulates genes that are protective against oxidative stress including those involved in glutathione synthesis and heme oxygenase 1 (HO‐1).^76^ It also suppresses transcription of proinflammatory genes, for example IL‐1β and therefore controls tissue injury during inflammation.^77^ Recent interesting work has demonstrated a role for NRF2 in orchestrating macrophage metabolism following an inflammatory stimulus, with NRF2 a core regulator of lipid and glutathione metabolism as well as promoting mitochondrial fusion.^78^ Out‐with the ability of NRF2 to regulate neutrophil cytokine production and migration^79^, ^80^ it will therefore be important to consider how neutrophils meet both their biosynthetic and anti‐oxidant needs through co‐ordinated responses in 1C metabolism and NRF2 signaling.

## 6 NUTRIENT‐SENSING KINASES: MTOR AND AMPK

**FIGURE 4:**
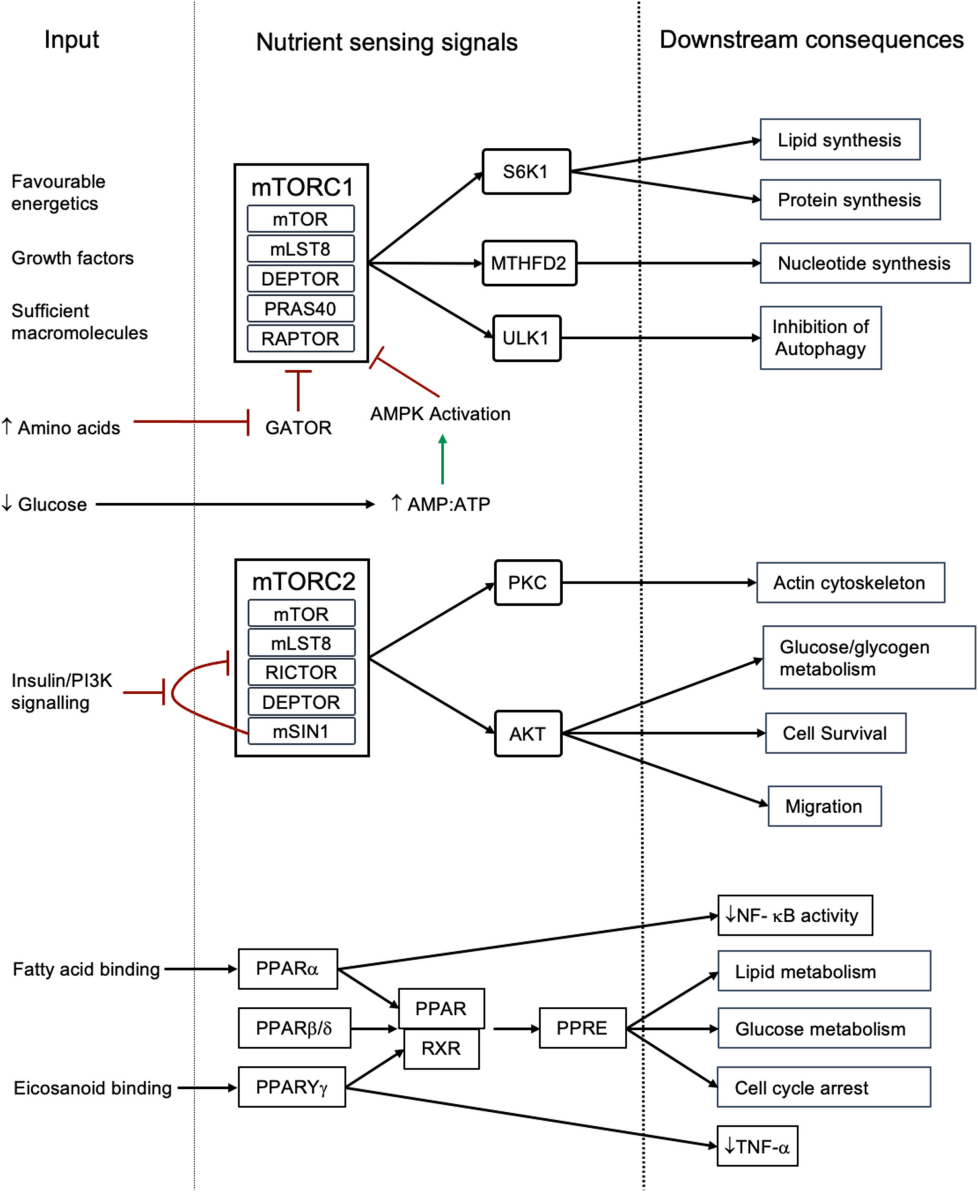
Nutrient‐sensing pathways integrate a multitude of intra‐ and extracellular signals in order to drive anabolic and catabolic pathways as appropriate to maintain homeostasis

The mechanistic target of rapamycin (mTOR) pathway is highly conserved between species and has been demonstrated to have an important role in cell growth, cancer, and immunity, including in the neutrophil inflammatory response.^81^ The mTOR pathway acts as an integrator of cellular nutritional status and metabolic behavior. mTOR itself is a serine/threonine protein kinase which forms two distinct signaling complexes, mTORC1 and mTORC2 (incorporating Raptor and Rictor, respectively).^81^ These complexes integrate multiple inputs to regulate anabolic and catabolic processes. For example, energetic stress following glucose deprivation results in increased AMP, activation of AMP‐activated protein kinase (AMPK), phosphorylation of Raptor and inhibition of downstream mTORC1 signaling. In contrast, amino acids confer an anabolic signal to the cell by inhibiting upstream inhibition of mTORC1 by GATOR. This permits mTORC1 to activate downstream targets including S6K by phosphorylation. The downstream effects of mTORC1 signaling include the positive regulation of mRNA translation, lipid synthesis, glucose utilization, and inhibition of autophagy.^82^ Additionally, mTORC1 plays a role in nucleotide synthesis by upregulation of methylenetetrahydrofolate dehydrogenase/cyclohydrolase (MTHFD2) via one carbon metabolism.^83^ In contrast, mTORC2 regulates the response to extracellular cues, particularly insulin. Here, the insulin receptor leads to PI3K activation and the generation of PIP3 which relieves mTORC2 autoinhibition by its mSin1 subunit. The downstream signaling consequences of mTORC2 are distinct from mTORC1 and include the activation of Akt which phosphorylates and negatively regulates the transcription factor FoxO1/3a, excluding it from the nucleus to regulate its downstream targets including enzymes involved in glucose and glycogen metabolism.^81^ Through regulation of PKC, mTORC2 also regulates cytoskeletal remodeling, migration and through FoxO1/3a, signaling inhibits apoptosis and drives cell cycle progression. Finally, there is a cross talk between mTORC1 and HIF‐1α where mTORC1 enhances HIF‐1α translation and therefore glycolytic behavior.^84^ Thus, the AMPK/mTOR pathway assimilates the nutritional status of the cell to regulate metabolism, growth, survival, and division (Figure 4).

While much is known of the importance of mTOR signaling in immune cell survival and function in general, less is understood of its role in neutrophils. However, an important role in regulating neutrophilic inflammation upon entry to nutritionally challenging sites has recently emerged. In a murine lung injury model, reduced access to glucose and oxygen led to suppressed mTORC1 activity in neutrophils.^5^ In doing so, neutrophils enhanced lysosomal catabolism of extracellular protein, as had been previously observed in cancer cell lines,^85^ and ultimately contributed to enhanced sickness outcomes.

Cellular nutritional status is also regulated in part by the peroxisome proliferator‐activated receptor (PPAR) pathway (Figure 4). Three of these ligand‐inducible transcription factors have been identified: PPARα, PPARβ/δ, and PPARYγ. These have distinct and overlapping tissue distribution and ligand specificity. Broadly, however, they behave similarly as promiscuous receptors binding a range of lipid‐derived ligands to regulate energy homeostasis.^86^ For example, PPARα is activated upon fatty acid binding, while the repertoire of PPARγ includes the eicosanoids. Ligand binding releases co‐repressors from PPAR, recruits co‐activators, and allows heterodimerization with retinoid X receptor (RXR) which then binds to peroxisome proliferator hormone response elements (PPRE) to begin transcription of genes included in lipid and glucose metabolism as well as promoting cell cycle arrest.^87^ The ability of PPAR to promote fatty acid and glucose oxidation and insulin sensitization has also led to the use of the therapeutic agonists, thiazolidinediones in the management of patients with type 2 diabetes mellitus.^88^ Beyond regulating the cellular response to lipids, the PPARs have emerged as important regulators of the inflammatory response. Treatment of macrophages with PPARγ agonists suppressed TNF‐α production while PPARα inhibited transcription of Nf‐κB targets.^89^ Moreover, PPAR has been shown to promote the formation of pro‐resolution M2 macrophages and attenuated the proinflammatory response to *Mycobacterium tuberculosis* while enhancing autophagic machinery, lipid catabolism and ultimately increasing *M. tuberculosis* killing.^90^ Similarly, administration of PPAR agonists favors an M2, pro‐resolving macrophage phenotype and attenuating tissue injury in immune‐mediated liver and kidney inflammation.^91^ Lipid availability and signaling through the PPAR pathway therefore plays an important role in regulating cellular metabolism and modulating inflammatory and antimicrobial responses.

Neutrophils have also been demonstrated to be regulated by the PPAR family. In a guinea pig model of acute LPS‐induced airway inflammation, treatment with the PPARγ agonist pioglitazone improved airway function and reduced neutrophil numbers in the airway.^92^ In patients with sepsis, PPARγ expression was increased and was responsible for impaired chemotaxis which was rescued by PPAR inhibition in vivo.^93^ PPARγ activation with Rosiglitazone was found to polarize infiltrating neutrophils in a mouse model of ischemic stroke with a favorable effect on inflammation resolution.^94^ PPARγ has also gathered attention given its role in the promotion of cellular apoptosis and ability to reduce neutrophil number although its precise role in neutrophil apoptosis remains unclear and is likely ligand‐specific.^95^ There therefore remains a need to further characterize the effect of lipid sensing on the neutrophil transcriptional program and inflammatory outcomes.

## 7 TRAINING THE NEUTROPHIL RESPONSE

Historically, the immune system has been broadly divided into cells of the innate and adaptive systems with a defining feature of the latter being memory for previous insults. Recent observations have challenged the dogma that cells of the innate immune system fail to mount adaptive responses. For example, enhancement of mononuclear phagocyte responses has been demonstrated following infection^96^ or vaccination^97^ and has led to the term trained immunity being coined. Initially described in plants,^98^ this phenomenon differs from classic immunological memory in that it is non‐specific, no gene rearrangement occurs and antigen‐specific clones are not generated.^99^ Instead, central imprinting of hematopoietic stem cells has been implicated in such responses in mammals.^100^ Moreover, in T‐cell and monocyte populations alterations in the epigenetic landscape are also described at sites of action and within the bone marrow compartment. These changes have been linked to the availability of metabolic intermediaries with known links to oxygen‐sensing responses exemplified by S‐2‐hydroxyglutarate, acetyl‐CoA, and fumarate.^101^, ^102^, ^103^

Less is known of such rewiring responses in neutrophils but nevertheless, persistent modification in their behavior following challenge has been described. Our group has demonstrated that, although systemic hypoxia exacerbated neutrophil‐mediated host injury in a murine model of acute pneumonia, prior exposure of animals to hypoxia was protective.^104^ Consistent with such findings in other immune cells, rewiring of cellular metabolism was key. Specifically, modulation of neutrophil glycolysis conferred host protection to damaging neutrophil responses and was regulated by the oxygen‐sensing HIF1 pathway^104^ as has been shown in other leucocytes.^105^ There is increasing evidence to support a role for training in neutrophil responses. Prior hypoxic exposure has also been demonstrated to be protective in the context of rat models of gut ischemic/reperfusion injury, where the cytokine TNF‐α primed neutrophils to maintain epithelial integrity.^106^, ^107^ Recently published data challenge the dogma that nuanced and trained neutrophil responses do not occur. A role for altered chromatin assembly and nucleosome positioning has been demonstrated in defining inflammatory neutrophil subsets in the blood of patients with systemic lupus erythematosus^108^ and in determining the altered fate and phenotype of neutrophils isolated from different mouse tissues.^109^ Interestingly, when exposed to beta‐glucan, a known inducer of trained immunity, murine neutrophils adopted an altered epigenome with modulation of their reactive oxygen metabolism which ultimately defined their antitumor response.^110^ With new evidence that maladaptive innate immune training and hypoxic lung inflammation are in part consequent upon altered bone marrow myelopoiesis^111^, ^112^ the potential that epigenetic programs can sustain reprogramming of neutrophil physiological responses within both the tissues and bone marrow compartment is an exciting possibility. How metabolic pathways could regulate these epigenetic programs remains speculative but, with JmjC‐containing histone demethylases and ten‐eleven translocation (TET) DNA demethylases requiring both oxygen and 2‐oxoglutarate for enzymatic function,^113^ a role for metabolism in the regulation of epigenetic modifications is likely.

## 8 NEUTROPHIL METABOLISM IN DISEASE

Not only are neutrophils essential for host pathogen defense but when they become dysregulated, they can contribute to significant host injury and illness. With the expanding knowledge base linking metabolic process with cellular function, attention is increasingly focussed on neutrophil metabolism and host outcomes in different disease states. Work over the last decade has expanded our understanding of neutrophil metabolism in disease including in respiratory disease, diabetes mellitus, and cancer.

### 8.1 Inflammatory lung disease

One of the greatest therapeutic challenges for treating tissue damage caused by neutrophilic inflammation remains how to selectively target dysfunctional neutrophilic inflammation while preserving key immune effector functions. Murine models of hypoxic acute lung injury have revealed that, in the tissues, neutrophils have the capacity to scavenge extracellular proteins to fuel central carbon metabolism and sustain de novo protein synthesis in the lung. Thus, they can adapt to the protein‐rich, glucose‐deprived environments pathognomonic of acute lung injury, a process regulated in part by activation of the lysosomal compartment and concurrent suppression of mTORC1.^5^ This capacity for tissue injury is further augmented by the ability of neutrophils to regulate glycogen stores. Activation of hypoxia signaling pathways consequent upon genetic loss or pharmacological inhibition of the dominant prolyl hydroxylase domain‐containing enzyme, PhD2, result in a glycolysis‐dependent increase in neutrophil persistence in the lung and associated lung injury in mice.^7^ These hyperinflammatory, glycolysis‐fueled neutrophils ultimately result in host‐mediated morbidity and mortality, independent of the pathogenicity of the bacterial or viral challenge.^104^, ^112^ In human disease, metabolic perturbations in the tissue neutrophil compartment have yet to be defined, although intrinsic reprogramming of core metabolic programs is observed within the circulating neutrophil compartment of patients with COVID‐19‐associated ARDS.^114^ An observed increase in intracellular glutamate levels in blood neutrophils raises the intriguing possibility that neutrophil capacity for substrate switching is pre‐determined, in advance of their recruitment into the tissues. In marked contrast to energy‐rich acute lung inflammation, chronic inflammatory lung diseases (typified by chronic obstructive pulmonary disease [COPD]) are associated with a failure of neutrophils to maintain energy stores secondary to impaired glycogen synthesis.^8^ Thus, the balance between supply and demand would appear crucial in defining inflammation outcomes in acute and chronic inflammatory disease states.

### 8.2 The neutrophil in diabetes mellitus

Patients with diabetes mellitus have reduced quantities of, or response to insulin. As a result, among a range of metabolic perturbations, they have variable levels of hyperglycemia. This leads to neutrophil dysfunction with patients suffering from both impaired pathogen responses and systemic inflammation. It is peculiar that, despite being predominantly glycolytic, neutrophils in the context of hyperglycemia have been shown to have reduced rates of glycolysis in both animal models and patients.^115^, ^116^ Moreover, they have reduced flux into and through the pentose phosphate pathway and ultimately have reduced NADPH as a result.^115^ Neutrophils may instead accumulate carbohydrate through the sorbitol pathway^117^, ^118^, ^119^, ^120^ which, through the action of aldose reductase, consumes NADPH. Moreover, hyperglycemia leads to impaired human neutrophil glutathione production and therefore enhances oxidative stress.^120^ Together, these effects result in reduced substrate for NOX and impairment of the antimicrobial response.

The reduction of glucose utilization also raises the possibility that neutrophils may be paradoxically energetically compromised in the context of diabetes. Indeed, inflammatory neutrophils from a diabetic rat model had reduced ATP content and depolarized mitochondria which were associated with a failure of autophagy.^121^ Mitochondrial dysfunction is a feature of diabetes mellitus more broadly, particularly in type 2 diabetes where there may be a reduction in oxidative phosphorylation and loss of mitochondrial plasticity.^122^ It may be challenging to draw parallels between cells that are classically insulin response such as muscle or adipose and those that are not. However, in lymphocytes from patients with type 2 diabetes mellitus where insulin regulates glucose uptake and function,^123^ mitochondrial dysfunction has been observed.^124^ In addition, while neutrophils are not insulin responsive in the classical sense, a reduction in complex 1 activity has been shown in the neutrophils of patients with type 2 diabetes.^125^ In an STZ‐induced mouse model of diabetes, kidney cells demonstrated reduced superoxide production and mitochondrial number, despite increased D17 mitochondrial DNA (mtDNA) deletions—a biomarker of cellular stress.^126^ Derangement of mitochondrial function impairs the ability of cells to use substrates effectively and to switch between substrate as fasting state necessitates, leading to incomplete fatty acid oxidation and accumulation of intracellular lipid that may exacerbate insulin resistance.^122^ Of interest, this mitochondrial stress and DNA damage leads to the release of mtDNA, activation of intracellular DNA signaling pathways, and the promotion of inflammatory responses via DAMP signaling, further contributing to insulin resistance.^127^

The paradoxical energy impairment of neutrophil in diabetes^121^ raises interesting questions as to the metabolic response and the effect of this. In radioactive decarboxylation studies, diabetic rat neutrophils had greater ^14^CO_2_ production when cultured with ^14^C‐palmitate suggesting they performed more fatty acid oxidation.^115^ This raises the intriguing possibility that neutrophils enhance oxidative metabolism to compensate for a reduction in glycolysis. However, why a cell that has adapted to function in a low glucose environment would switch away from glucose when it becomes more freely available remains unknown. Moreover, the consequences of such alterations in mitochondrial metabolism for neutrophil function remain uncertain and is deserving of further study.

### 8.3 Neutrophils in the neoplastic niche

Beyond sterile and septic inflammation, neutrophil metabolism has also been demonstrated to play a role in cancer outcomes. In particular, neutrophils of an immature phenotype were shown to use oxidative metabolism of amino acids to support NET formation under conditions of glucose deprivation, ultimately leading to metastasis in a mouse breast cancer model.^128^ In the same 4T1 murine tumor model, adoption of mitochondrial oxidative metabolism and NADPH production supported the ROS production necessary to inhibit antitumoral immunity.^129^ Finally, alternative lipid use by myeloid‐derived suppresser cells was found to be oncogenic where fatty acid transport protein (FATP)2 promoted lipid uptake and prostaglandin E2 synthesis with the suppression of the antitumor immune response.^130^ Thus, through metabolic reprogramming, neutrophil function may be regulated with direct consequences for the host. It is also interesting to note that, in the innate immune training of tumor‐bearing mice, granulocyte‐monocyte precursors enrich at a pathway level for transcripts related to oxidative phosphorylation, mitochondrial dysfunction, and mTOR signaling, hinting that metabolic adaptations within myeloid progenitors in the bone marrow may in part underlie this trained response.^110^ Further complexity is introduced by the heterogeneity of neutrophil populations observed in the setting of cancer. Low density and normal density, mature and immature cells contribute to the circulating pool and different subsets may be recruited to primary versus metastatic tumor sites. It remains to be clarified whether this diversity is in part consequent upon metabolic specialization of these different neutrophil compartments and how this informs pro‐ or antitumor neutrophil responses. It is, however, reported that these different neutrophil populations have a different propensity to produce ROS and undergo NETosis; thus, a better understanding of the mechanisms which regulate neutrophil metabolism may provide important clues for therapeutic targets in neoplastic disease states.

## 9 SUMMARY

**FIGURE 5:**
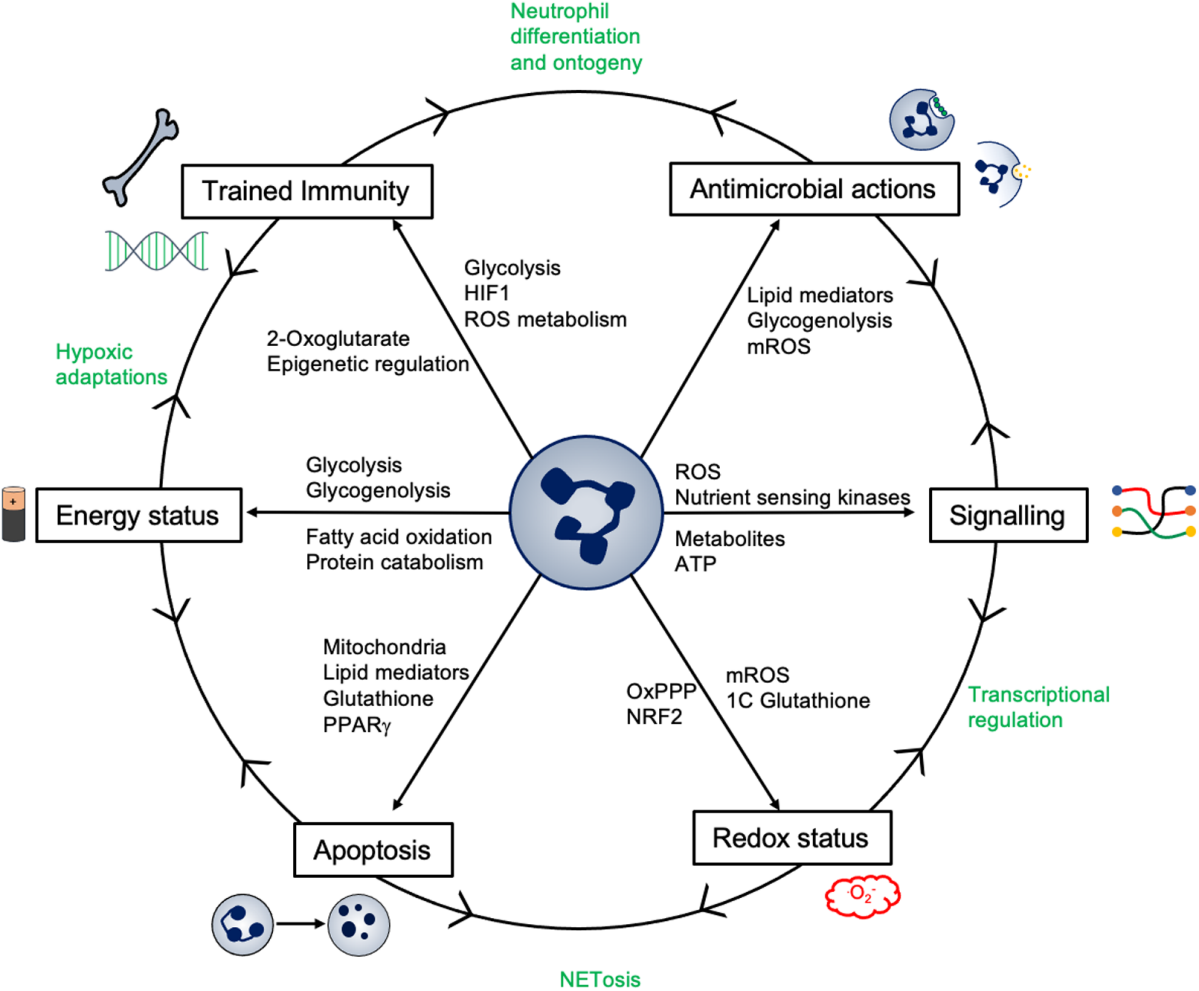
Neutrophil metabolism is dynamic and flexible. Regulation of metabolic pathways has implications for a multitude of functions including energetics, phagocytosis, redox status, signaling survival, differentiation, and trained immunity. Many important neutrophil functions are consequent upon a combination of these factors, illustrated in green

Neutrophils are a heterogenous population of dynamic effector cells of the innate immune response that have evolved to function in conditions of limited oxygen and nutrient availability. They are metabolically specialized cells, a consequence of their short‐lived nature, and need to rapidly access ATP to mount an effective pathogen response in hostile sites. These adaptions while crucial for pathogen control can be subverted in disease states resulting in a cycle of tissue injury which is self‐sustained and difficult to break. This review highlights the many facets of neutrophil metabolism which contribute to their inflammatory capacity, including the role of mitochondria, despite a dependence on glycolysis and the many aspects of lipid metabolism which instruct neutrophilic functions. We also discuss the roles of nutrient‐sensing pathways and 1C metabolism, previously thought to be less relevant to the non‐proliferative neutrophil but now understood to play an important role determining phenotype and function in these cells. Through better understanding of the processes that underpin the metabolic peculiarity of these cells, there is the exciting potential to identify targets that are specific to the neutrophil, retained over time and independent of effects on essential antimicrobial effector functions (Figure 5). In this regard, there remains a significant knowledge gap in where aberrant neutrophil responses originate and how they relate to changes in metabolite utilization extending well beyond their capacity to generate ATP. This includes but is not limited to the ability of metabolic programs to regulate neutrophil apoptosis programs, NETosis, redox balance, membrane turnover, biosynthetic programs, inflammatory signaling cascades, epigenetic marks, trained immunity, and developmental programs of both mature neutrophils and their progenitors.

## CONFLICT OF INTEREST

The authors have no conflict to declare.

## Data Availability

Data sharing is not applicable to this article as no new data were created or analyzed in this study.
